# Evidence That Anemia Accelerates AS Progression Via Shear-Induced TGF-β1 Activation: Heyde's Syndrome Comes Full Circle

**DOI:** 10.1016/j.jacbts.2023.09.007

**Published:** 2023-11-01

**Authors:** Kumar Subramani, Jeffrey Bander, Sixia Chen, Mayte Suárez-Fariñas, Thamizhiniyan Venkatesan, Sandeep Subrahmanian, Rohan Varshney, Annapoorna Kini, Samin Sharma, Daniel B. Rifkin, Jaehyung Cho, Barry S. Coller, Jasimuddin Ahamed

**Affiliations:** a Cardiovascular Biology Research Program, Oklahoma Medical Research Foundation, Oklahoma City, Oklahoma, USA; b Icahn School of Medicine at Mount Sinai New York, New York, USA; c University of Oklahoma Health Sciences Centers, Oklahoma City, Oklahoma, USA; d Departments of Cell Biology and Medicine, New York University, New York, New York, USA; e Washington University School of Medicine, St. Louis, Missouri, USA; f Laboratory of Blood and Vascular Biology, Rockefeller University, New York, New York, USA

**Keywords:** anemia, aortic stenosis, AVWS, GI bleeding, Heyde’s syndrome, TGF-beta

## Abstract

The severity of aortic stenosis (AS) is associated with acquired von Willebrand syndrome (AVWS) and gastrointestinal bleeding, leading to anemia (Heyde's syndrome). We investigated how anemia is linked with AS and AVWS using the LA100 mouse model and patients with AS. Induction of anemia in LA100 mice increased transforming growth factor (TGF)-β1 activation, AVWS, and AS progression. Patients age >75 years with severe AS had higher plasma TGF-β1 levels and more severe anemia than AS patients age <75 years, and there was a correlation between TGF-β1 and anemia. These data are compatible with the hypothesis that the blood loss anemia of Heyde's syndrome contributes to AS progression via WSS-induced activation of platelet TGF-β1 and additional gastrointestinal bleeding via WSS-induced AVWS.

## Highlights

• Older AS patients and LA100 mice (AS mouse model) have higher plasma TGF-β1 levels and more severe anemia than younger AS patients and LA100 mice.
• Anemia is linked to progression of AS and AVWS in a mouse model and patients with AS.
• The blood loss anemia in Heyde's syndrome itself may lead to AS progression through increasing WSS and activation of TGF-β1, bringing Heyde's syndrome full circle.
• Plasma TGF-β1 levels and AVWS may be valuable biomarkers of AS progression and treatment of anemia may be an intervention worthy of study.

## Visual Abstract

**Figure:**
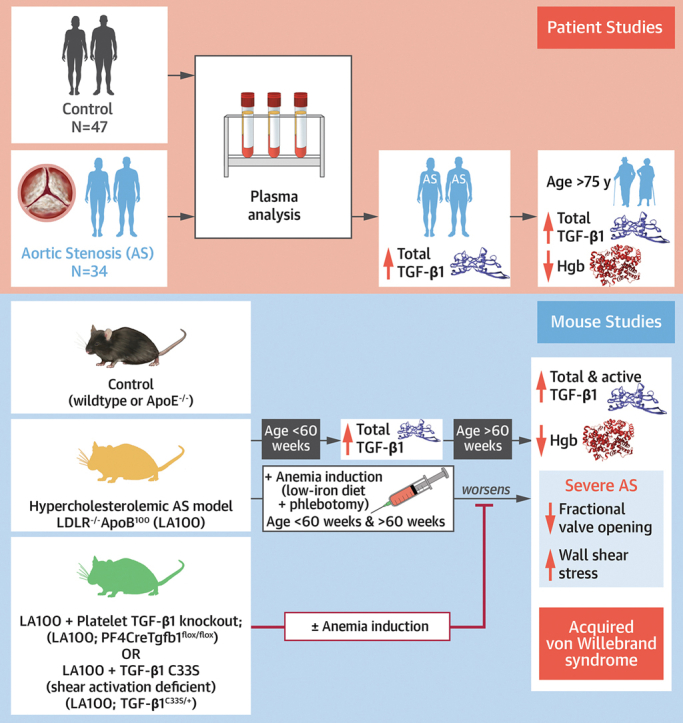

Aortic stenosis (AS) is a progressive disorder, and so the severity increases with age, with 4% to 13% of individuals over the age 75 years having severe AS.^1^^,^^2^ As the U.S. population ages, the prevalence of AS is expected to increase dramatically. AS is a degenerative chronic progressive disorder characterized by thickening of the valve leaflets caused by fibrosis and calcification, leading to narrowing of the aortic orifice with obstruction of left ventricular outflow.^3^ Narrowing of the valve opening results in increased wall shear stress across the valve during systole, increased cardiac preload, ventricular hypertrophy, and myocardial fibrosis, which ultimately result in heart failure, syncope, and sudden death.^4^^,^^5^ In 1958, Heyde described an association between severe AS and gastrointestinal bleeding leading to anemia, and subsequently the bleeding disorder was ascribed to the development of an acquired form of von Willebrand syndromes (AVWS) in which increased wall shear stress across the aortic valve results in a conformational change in the von Willebrand factor (vWf), resulting in cleavage of vWf by the plasma protease a disintegrin and metalloproteinase with a thrombospondin type 1 motif, member 13 (ADAMTS13) and loss of the more hemostatically active high molecular weight vWf multimers.^6^, ^7^, ^8^ AVWS has been described in 67% to 92% of patients with severe AS, with the severity of the abnormality in vWf being directly related to the severity of AS. Notably, AVWS in AS patients is reversed after valve replacement.^9^ Because a diagnosis of AVWS requires specialized testing, Heyde’s syndrome is likely underdiagnosed in AS patients.^7^^,^^10^ Anemia itself has been reported to be associated with more severe AS as well as increased all-cause mortality in medically treated AS patients.^11^ Mild anemia was observed in AS patients older than 75 years of age, and more severe anemia was observed in AS patients older than 80 years,^11^ the age range in which Heyde’s syndrome is more prevalent.^7^^,^^11^ The negative impact of anemia on AS prognosis has been ascribed to the direct and indirect effects of anemia on oxygen delivery, left ventricular hypertrophy, and cardiac fibrosis.^12^^,^^13^ However, the mechanistic link between anemia and AS progression is not clear.

Transforming growth factor (TGF)-β1 is a multifunctional cytokine that affects many biological and pathological processes, including cell proliferation and differentiation, the immune response, and pathological tissue fibrosis.^14^ Polymorphisms in the TGF-β1 gene have been associated with AS,^15^ and higher plasma levels of TGF-β1 have been reported in severe AS patients before undergoing valve replacement surgery.^16^ In addition to profound profibrotic effects, TGF-β1 has also been shown to induce calcification in explanted cardiac valve cells in vitro.^17^ Platelets contain 40 to 100 times more TGF-β1 than other cell types and rapidly release TGF-β1 upon activation.^18^^,^^19^ Platelets release TGF-β1 as a latent complex in which mature TGF-β1 (25 kD) is noncovalently bound to latency-associated peptide (LAP), and LAP forms a disulfide bond via its cysteine residue 33 (C33) with latent TGF-β binding protein 1 (LTBP-1).^20^ Shear stress activates latent TGF-β1 released from platelets in vitro, and the binding of LAP to LTBP-1 is critical for shear stress-induced TGF-β1 activation.^18^ Thiol-disulfide exchange partially contributes to shear stress-dependent latent TGF-β1 activation, and protein disulfide isomerase (PDI) likely facilitates thiol-disulfide exchange during shear stress-induced TGF-β1 activation.^21^

Mice with targeted inactivation of TGF-β1 in their megakaryocytes and platelets are partially protected from developing cardiac hypertrophy, fibrosis, and systolic dysfunction in a pressure overload transverse aortic constriction model,^22^ and are partially protected from AS progression in a murine hypercholesterolemic model (low-density lipoprotein receptor [LDLR]^−/−^ apolipoprotein [Apo]B^100^; LA100) in which mice spontaneously develop AS.^22^^,^^23^ Because platelets can also be activated by wall shear stress,^24^ AS may activate platelets to release latent TGF-β1, and the latter can subsequently be activated by wall shear stress, creating a vicious cycle that contributes to AS progression.

We hypothesized that anemia may bring Heyde’s syndrome full circle, potentiating the progression of AS by further increasing wall shear stress, leading to release and activation of platelet-derived TGF-β1. To test this hypothesis, we evaluated plasma TGF-β1 levels in patients with severe AS as a function of anemia and age, and correlated the results with studies of TGF-β1 in animal models of AS with, and without, artificially induced anemia.

## Methods

### Patient population

Informed consent was obtained from all participants in accordance with a protocol approved by the Rockefeller University, Mount Sinai Medical Center, and Oklahoma Medical Research Foundation Institutional Review Boards. Patients with severe AS (n = 35) were recruited from Mount Sinai Medical Center based on either a mean aortic valve pressure gradient ≥40 mm Hg and/or a calculated aortic valve area ≤1 cm^2^. A separate patient control group (n = 47) consisted of patients with cardiovascular disease undergoing cardiac catheterization or an electrophysiological study who did not have severe AS by echocardiography. Healthy control subjects (n = 20) without a history of heart disease were recruited at Rockefeller University and Oklahoma Medical Research Foundation.

### Assessment of AS in human patients by echocardiography and cardiac catheterization

2-dimensional transthoracic echocardiograms and cardiac catheterization were performed on AS patients and patient control subjects. Transaortic valve gradients were calculated by the modified Bernoulli equation or by direct measurement during cardiac catheterization. Aortic valve orifice areas were calculated either by the continuity equation from transthoracic echocardiogram data or the Gorlin equation from cardiac catheterization data.^25^

### Preparation of human plasma samples

Blood samples were collected in vacuum tubes containing sodium citrate (Vacutainer, BD BioSciences) and immediately centrifuged at 12,000 *g* for 5 minutes at 4 °C to prevent in vitro release of TGF-β1 from platelets as previously described.^26^ As an additional quality control measure to eliminate samples that had undergone in vitro release of a-granule contents, plasma samples were analyzed by immunoblot for the platelet-specific proteins PF4 and TSP-1, which are contained in α-granules. Plasma samples from healthy control subjects prepared immediately after blood drawing had low TGF-β1 levels (1-3 ng/mL) and nearly undetectable levels of PF4 and TSP-1 antigen.^26^ Based on this data, we excluded samples with elevated levels of TSP-1 and PF4 from analysis. We did not exclude samples with isolated increases in TSP-1 because this pattern likely reflected in vivo release, as PF4 released from platelets in vivo binds to endothelial cell glycosaminoglycans.^27^^,^^28^

### Murine models

Murine models include the following: 1) hypercholesterolemic mice lacking low-density lipoprotein receptors (LDLR^−/−^) and only expressing ApoB-100 allele (LDLR^−/−^ApoB^100^, B6;129S-*Ldlr*^*tm1Her*^ *Apob*^*tm2Sgy*^/J, Jackson strain #003000; LA100); 2) ApoE^−/−^ mice, which develop atherosclerosis, *Apoe*^*tm11unc/*^*J*, Jackson strain #002052; 3) control WT-C57BL/6 mice of both sexes; 4) LA100 mice crossed with C33S^+/−^ mice whose latent TGF-β1 cannot be activated by shear force^18^ to obtain LA100;C33S^+/−^ mice (Supplemental Figure 1A); 5) LA100;PF4CrePDI^flox/flox^ mice generated by crossing LA100 mice with PF4CrePDI^flox/flox^ mice (Supplemental Figure 2); and 6) LA100;PF4CreTgfb1^flox/flox^ mice, previously described.^23^ All mice were maintained on a chow diet for up to 24 months. Blood samples from all mice were drawn by the retro-bulbar (RB) puncture method into tubes containing 0.1 volume 3.8% sodium citrate, pH 7.4, 1 μmol/L prostaglandin E_1,_ as previously described.^22^ All murine experiments were compliant with standards of Guide for Care and Use of Laboratory Animals of the National Institute of Health and approved by the Animal Care and Use Committees at Oklahoma Medical Research Foundation. Both male (50%-60%) and female (30%-40%) mice were used for the experiments.

### Iron deficiency anemia mouse models

LA100, WT-C57Bl/6, and PF4CreTgfb1^flox/flox^ mice were fed an iron-deficient diet (2-6 ppm iron [TD.80396; Harlan Laboratories] compared with a normal diet containing ∼270 ppm iron). Blood (<100 μL) was obtained from the mice before initiation of the iron-deficient diet (baseline or 0 week) and then they were phlebotomized of 300 to 500 μL of blood by RB puncture thereafter at weeks 1 (phlebotomy 2), 3 (phlebotomy 3), 5 (phlebotomy 4), 6 (phlebotomy 5), 8 (phlebotomy 6), 9 (phlebotomy 7), and 11 (phlebotomy 8). Control mice for these experiments were LA100 mice that underwent low-volume phlebotomy (100 μL) before (pre) and again at week 11. For mild to moderate anemia, LA100 or LA100;PF4CreTgfb1floxed mice were fed with low-iron diet (2-6 ppm iron [TD.80396; Harlan Laboratories]) for 8 weeks without phlebotomy. Blood from these mice was collected by RB puncture into tubes containing 0.1 volume 3.8% sodium citrate, 1 μmol/L PGE_1,_ as described previously,^22^ and blood counts were assessed using an automated dual-angle laser detection system (HEMAVET HV950; Drew Scientific, Inc). The citrated blood was centrifuged at 12,000 *g* for 5 minutes at room temperature immediately after blood drawing, and the plasma was frozen at −80 °C until assayed for TGF-β1.

### Assessment of AS in mouse models by echocardiography

Ultrasound echocardiography imaging was obtained for clear images of aortic valve cusps; an aortic arch view was obtained using a modified method (Supplemental Figure 3A, bottom). We also used an improved method to measure peak blood flow velocity across the valves (jet) with a combination of color and pulse-wave Doppler that allows alignment along the maximum flow direction (Supplemental Figure 3A, top), as described in detail in our previous study.^23^ Our echocardiography method directly measures both the area and velocity and is relatively easy to perform even in mice with much faster heart rates than humans.

### Tissue processing and whole mount staining for light, confocal, and scanning electron microscopy imaging

Whole aortic valve tissues were fixed in 4% paraformaldehyde and pinned along with surrounding tissues on a dissecting petri dish using a high-power surgical microscope to open and flatten the aortic valve leaflets. Tissues were stained with antibodies to the platelet-specific protein PF4 and the RBC-specific protein TER119 and were imaged using a Ziess 710 confocal microscope to obtain z-stacks and tiled pictures for colocalization analysis. Valve tissues were processed for scanning electron microscopy imaging using a FEI Quanta 600 electron microscope, as previously described.^23^

### Measurement of TGF-β1

Total TGF-β1 in human and mouse plasma was measured with an antibody enzyme-linked immunosorbent assay (ELISA) specific for the activated form of TGF-β1 (R&D Systems) after converting latent TGF-β1 to active TGF-β1 by acidification (20 minutes incubation at RT with 0.5 volume of 1 N HCl, followed by neutralization with the same volume of 1.2 N NaOH in 0.5 mol/L HEPES), as described previously.^18^ Active TGF-β1 was measured by directly applying plasma without acid activation and dilution using the same ELISA kit.

### vWf multimers analysis

Plasma vWf multimers were analyzed by electrophoresing plasma samples in discontinuous 1.5% agarose gels, followed by immunoblotting with an anti-vWf monoclonal antibody and detection by imaging and densitometry. High molecular weight (HMW) vWf multimers were defined as the area under the curve of all multimers above the 7th multimer band (starting from the bottom) expressed as a percentage of the area under the curve of all multimer bands as described previously.^26^

### Statistical analysis

Statistical calculations were performed using SPSS 20 (International Business Machines Corp) and R version 2.1.12. For comparisons between case and control subjects, demographic characteristics were presented using the mean ± SD or median with 25th and 75th percentiles (Q1, Q3) for continuous variables and count (percentage) for dichotomous variables. Continuous variables were compared using Student's *t*-test for normally distributed data and the Mann-Whitney *U* test for non-normally distributed data. The Shapiro-Wilk test was used to assess whether the variables initially followed the normal distribution. Categorical variables were compared using the Fisher exact test. Linear mixed-effects models were used to compare changes over time across different groups. Specifically, we used mixed-effect models with time, group, and its interactions as fixed effects and a random intercept for each mouse, under a compound symmetric correlation structure. Comparisons of interest were assessed through contrasts, and the estimated marginal means and SEM are presented in summary plots. Correlation among continuous values was assessed using Pearson's correlation coefficient (r) or linear regression analysis. Because TGF-β1 levels were measured by enzyme-linked immunosorbent assay and the values were log normally distributed, we converted all values to log_10_ for analysis. Data are shown as median (Q1, Q3), mean ± SD, or mean ± SEM. Multivariable linear regression models were used to evaluate TGF-β1 association with other variables, such as hemoglobin (Hb) levels, age, and gender as covariates. Variable selection was carried out using backward stepwise regression, and the optimal model was based on the Akaike information criteria (AIC).^29^ For the risk model, a logistic regression was fitted with the factors of interest through a generalized linear model and a similar variable selection procedure was applied. ORs and 95% CIs were estimated from this model. A 2-sided *P* value <0.05 was considered signiﬁcant. Correlation coefficients referred to are Pearson’s correlation unless otherwise specified. Results for Spearman’s correlation were largely the same, as presented in the Results section.

For mouse samples, the statistical analysis was performed using SAS version 9.4 (SAS Institute, Inc). We first calculated log_10_ transformation for total TGF-β1 and active TGF-β1 values. Then, for the transformed total TGF-β1 and active TGF-β1 variables as well as Hb, cusp separation, fractional valve opening, and WSS, we first used linear mixed effects models to examine their association with bleeding time or scan time, age, Hb, and the corresponding interaction terms. We used the multiple linear regression model for total TGF-β1, active TGF-β1, and Hb. Collinearity among predictors was examined. In addition, we also fit separate models for 2 age groups. For cusp separation, fractional valve opening, and WSS, the Kruskal-Wallis test and pairwise 2-sample Wilcoxon rank sum tests were used.

For original data, please contact the corresponding author.

## Results

### Patient demographics and hemodynamic measurements

Data (demographic and categorical) on the 47 patient control subjects and 34 AS patients are provided in Table 1. The AS patients were approximately 10 years older than the patient control subjects (age 63 ± 14 years vs 75 ± 10 years) and less likely to be taking clopidogrel, but they did not differ in any other measured characteristics. AS patient hemodynamic data and calculated aortic valve measurements are summarized in Table 1.

**Table 1:** Patient Demographics

	Patient Control Subjects	AS Patients	*P* Value
Age, y	63 ± 14	75 ± 10	<0.001a
Glomerular filtration rate, mL/min	61.1 ± 13.4	61.8 ± 15.5	0.90b
Hemoglobin, g/dL	12.8 ± 1.5	12.5 ± 1.8	0.41a
Platelet count, x10^3^/μL	184 ± 47	193 ± 76	0.64b
Left ventricular ejection fraction, %	54 ± 12	59 ± 13	0.07b
Female	17 (36)	18 (53)	0.17
Hypertension	34 (76)	21 (70)	0.61
Diabetes mellitus	12 (27)	7 (23)	0.80
Coronary artery disease	22 (73)	16 (80)	0.74
Statin use	26 (57)	19 (58)	1.00
Angiotensin receptor blocker use	10 (22)	7 (22)	1.00
Angiotensin-converting enzyme inhibitor use	11 (23)	8 (24)	1.00
Beta-blocker use	27 (57)	19 (58)	1.00
Coumadin use	5 (10)	7 (21)	0.22
Aspirin use	33 (70)	22 (67)	0.81
Clopidogrel use	17 (36)	5 (15)	0.050
Aortic valve parameters among patients with AS		(n = 34)	
Peak gradient, mm Hg; TTE		69 ± 25	
Mean gradient, mm Hg; TTE		41 ± 15	
Calculated aortic valve area, cm^2^		0.75 ± 0.15	
Mean aortic valve shear stress, dynes/cm^2^^c^		94 ± 25	

Values are mean ± SD or n (%). Statistical analysis performed by either ^a^Student’s *t*-test or ^b^Mann-Whitney *U* test.

AS = aortic stenosis; TTE = transthoracic echocardiography.

c Shear stress calculated according to the formula of Vincentelli et al.^9^

### Plasma levels of total TGF-β1 in AS patients were higher than patient control subjects and correlate with age

**Figure 1:**
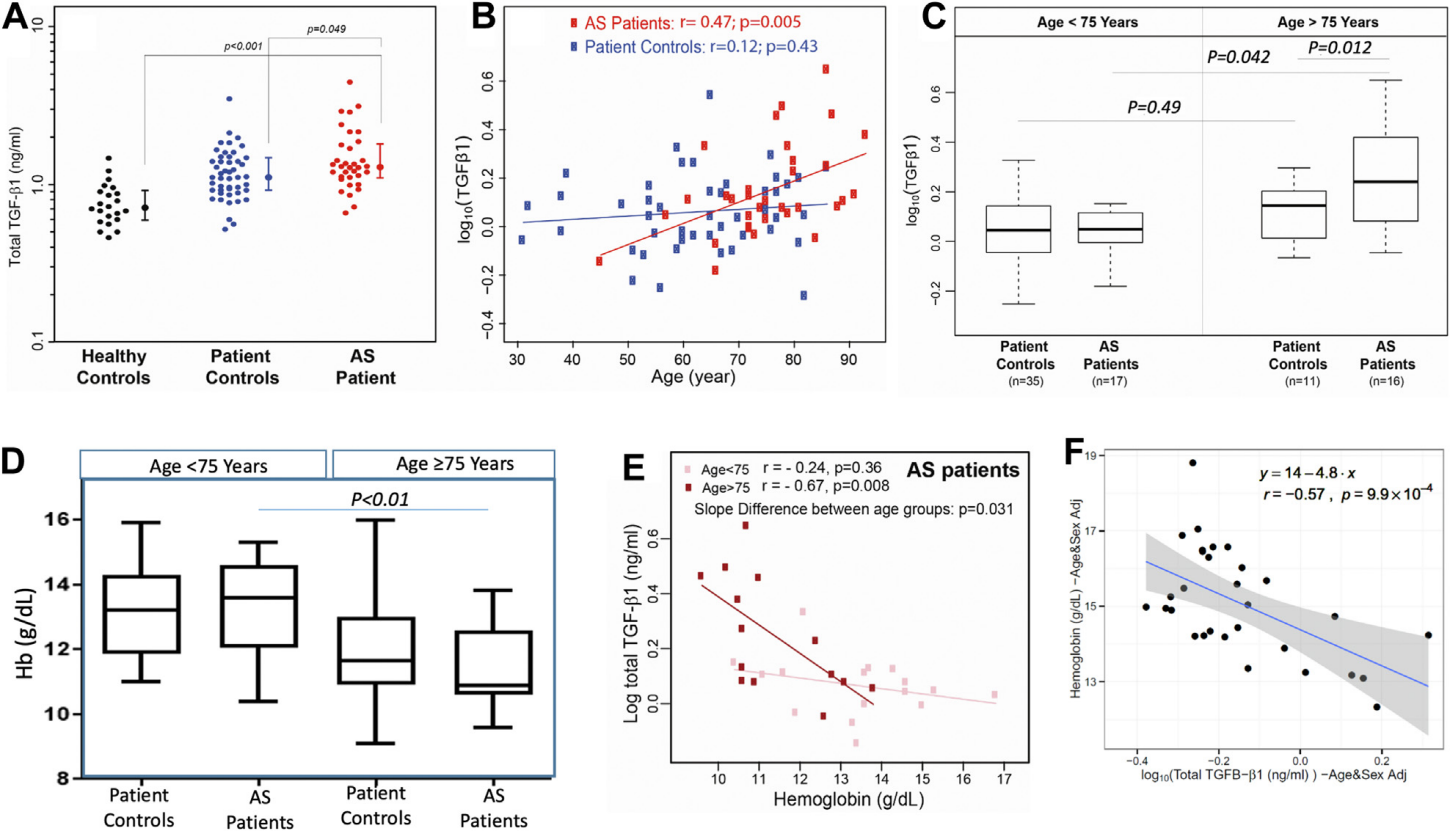
AS Patients Have Higher Plasma Levels of Total TGF-Β1 Than Patient Control Subjects and TGF-Β1 Levels Correlate With Age in AS Patients (A) Total transforming growth factor (TGF)-β1 levels in plasma samples from healthy control subjects, patient control subjects, and aortic stenosis (AS) patients were measured by enzyme-linked immunosorbent assay. Individual subjects are shown as filled circles and plotted on a log scale with the median (Q1, Q3) shown as box and whisker plots. (B) Plasma TGF-β1 levels were positively correlated with age in AS patients (r = 0.47; *P =* 0.005), but not in patient control subjects (r = 0.12; *P =* 0.43). The *P* value for the group and age interaction in the analysis of variance model was 0.030, indicating a significant difference in the slope of the regression lines. (C) Total TGF-β1 levels in plasma of AS patients (n = 16) and patient control subjects older than 75 years of age (n = 11), and AS patients (n = 17) and patient-control subjects (n = 35) younger than 75 years of age. The older AS patients had higher levels than the older patient control subjects *(P =* 0.012) and the younger AS patients *(P =* 0.042). (D) Hemoglobin (Hb) levels in AS patients and patient control subjects older and younger than 75 years of age. (E) Correlation between TGF-β1 and Hb levels in AS patients >75 years and <75 years. (F) Correlation between TGF-β1 and Hb levels in AS patients >75 years after adjusting for age and sex.

Plasma samples were prepared and 1 AS patient sample was excluded from analysis based on the quality control criteria we described previously.^26^ Plasma levels of total TGF-β1 were significantly higher in AS patients compared with healthy control subjects or patient control subjects (Figure 1A) (Median plasma levels of total TGF-β1 were significantly higher in AS patients [1.29 ng/mL (Q1, Q3: 1.10, 1.81 ng/mL], n = 34) compared with patient control subjects [1.10 ng/mL (Q1, Q3: 0.92, 1.48 ng/mL), n = 47; *P =* 0.049] and healthy control subjects (0.71 ng/mL (Q1, Q3: 0.60, 0.91 ng/mL), n = 22; *P <* 0.001]). Age is a known risk factor for AS development,^30^ and in our study, the AS patients were older than the patient control subjects (AS: 75 ± 2.6 years vs 65 ± 16 years; *P* < 0.001). Age was positively correlated with total TGF-β1 levels in AS patients (r = 0.475; *P =* 0.005), but not patient control subjects (r = 0.12; *P =* 0.43) (Figure 1B).

### AS patients older than 75 years of age have higher levels of plasma total TGF-β1 and are more anemic than AS patients younger than 75 years of age

To test the possibility that age could be a confounding factor for the elevated levels of TGF-β1 observed in AS patients, we compared TGF-β1 levels in patients older than 75 years with those 75 years of age or younger (≤75 years). We found that the older AS patients had significantly higher median levels of plasma TGF-β1 (1.74 ng/mL [Q1, Q3: 1.20, 2.76 ng/mL], n = 16) compared to older patient control subjects (1.40 ng/mL [Q1, Q3: 0.98, 1.60 ng/mL], n = 11; *P =* 0.012] and younger AS patients (1.20 ng/mL [Q1, Q3: 0.97, 1.32 ng/mL], n = 17; *P =* 0.042) (Figure 1C). No difference was observed in TGF-β1 levels between age groups in patient control subjects.

We also found that older AS patients had lower Hb levels than younger AS patients (Figure 1D) (*P <* 0.010). No difference in Hb levels was observed between the older and younger patient control subjects (*P =* 0.57).

### TGF-β1 is inversely correlated with Hb in older AS patients and is associated with AS progression

We also observed an inverse correlation between plasma TGF-β1 levels and Hb in older AS patients (Figure 1E) (r = −0.67; *P =* 0.008), but not younger patients. The inverse correlation between plasma TGF-β1 levels and Hb in older AS patients remained significant even after adjusting for age and sex (r = −0.57; *P <* 0.01) (Figure 1F).

Using a logistic regression model in older AS patients (age >75 years), factors found independently associated with AS were increasing age (OR: 1.32; *P =* 0.020) and total TGF-β1 levels (OR: 4.08; *P =* 0.020). In terms of TGF-β1 levels, this OR means that going from 1 to 1.5 ng/mL (roughly the first and third quartiles of the observed values for TGF-β1), the risk increases 2.88-fold. Age also was a risk factor in patients age ≤75 years; age was still associated with AS (OR: 1.13; *P* < 0.001) but not TGF-β1. A unified risk model combining all patients with terms for the interaction among age, TGF-β1, and Hb resulted in similar conclusions.

### Hypercholesterolemic, LDLR^−/−^ApoB^100^ (LA100) mice spontaneously develop AS at older ages and have elevated plasma levels of TGF-β1 compared with control mice

**Figure 2:**
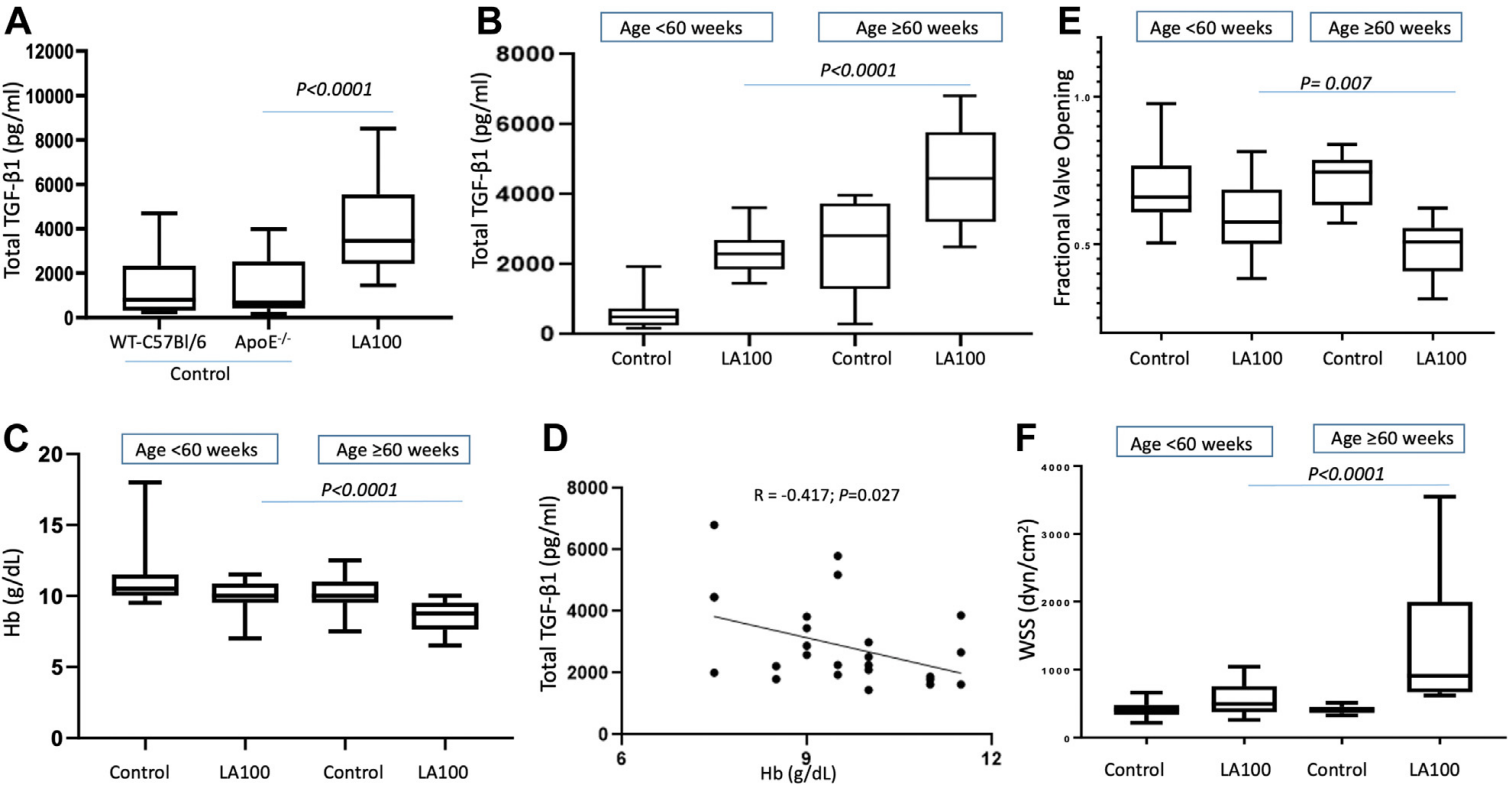
LA100 Mice, Which Spontaneously Develop AS, Have Higher Plasma Total TGF-β1 and Lower Hb Levels Than Control Mice (A) Plasma samples from control WT-C57Bl/6 and ApoE^−/−^ mice (both do not develop AS; 25-125 weeks of age) and age-matched LA100 mice were assessed for total TGF-β1 levels by enzyme-linked immunosorbent assay. (B) Total TGF-β1 levels in plasma of older (>60 weeks) and younger (<60 weeks old) LA100 and control mice. Older LA100 mice had higher TGF-β1 than younger LA100 mice or older control mice (*P <* 0.0001) (C) Hb levels in older and younger LA100 and control mice. Older LA100 mice have lower Hb levels than younger mice (*P <* 0.0001) (D) Correlation between TGF-β1 and Hb levels in older LA100 mice (r = −0.417; *P =* 0.027). (E, F) AS progression parameters fractional valve openings and wall shear stress (WSS) measured by echocardiography in control and LA100 mice <60 and >60 weeks of age. Abbreviations as in Figure 1.

To assess whether plasma TGF-β1 was also elevated in murine models of AS, blood was collected from hypercholesterolemic LA100 mice which spontaneously develop AS with aging, most commonly after approximately one year. We found that average plasma TGF-β1 levels in LA100 mice (4.0 ± 1.3 ng/mL, n = 63, ages 25-125 weeks) were significantly higher than those in age-matched hyperlipidemic ApoE−/− (1.4 ± 1.3 ng/mL, n = 30; *P* < 0.001) and WT-C57BL/6 control mice (1.45 ± 1.4 ng/mL, n = 39; *P* < 0.001) (Figure 2A).

### LA100 mice older than 60 weeks of age have higher levels of plasma total TGF-β1 and lower Hb than LA100 mice younger than 60 weeks of age

To assess age effects in the murine model of AS, we compared TGF-β1 levels in mice older than 60 weeks (>60 weeks; n = 25) vs those 60 weeks of age or younger (≤60 weeks; n = 26). Considering that both ApoE^*−/−*^ and WT C57BL/6 mice do not develop AS and there was no significant difference in plasma total TGF-β1 levels between groups, both mouse cohorts served as a pooled control group. We found that LA100 mice >60 weeks had higher levels of plasma TGF-β1 compared with LA100 mice ≤60 weeks (4.4 ± 1.4 vs 2.5 ± 0.6, respectively; *P <* 0.001) (Figure 2B). LA100 mice >60 weeks of age (n = 44) had lower median Hb levels than younger LA100 mice (n = 24) (8.8 g/dL [Q1, Q3: 7.8, 9.5 g/dL] vs 10.0 g/dL [Q1, Q3: 9.5, 10.6 g/dL], respectively; *P <* 0.001) (Figure 2C). Control mice >60 weeks of age (n = 38) had lower median Hb levels compared with control mice <60 weeks of age (n = 31) (10.0 g/dL [Q1, Q3: 9.5, 10.6 g/dL] vs 10.5 g/dL [Q1, Q3: 10.0, 11.4 g/dL], respectively; *P =* 0.040) (Figure 2C). Total TGF-β1 levels negatively correlated with Hb in LA100 mice >60 weeks of age (r = −0.417; *P =* 0.027) (Figure 2D).

### LA100 mice >60 weeks of age developed severe AS whereas control mice did not

LA100 mice >60 weeks had more severe AS (n = 34) as measured by decreased fractional valve opening (calculated by dividing cusp separation distance by left ventricular outflow tract distance) as previously described^23^ (Figure 2E), and increased wall shear stress (Figure 2F) compared with younger LA100 mice (n = 17). No AS was observed in control mice (ApoE^−/−^ or C57Bl/6), even at ages over 100 weeks (n = 17-49) (Figures 2E and 2F).

### Induced severe iron deficiency anemia in LA100 mice leads to higher levels of plasma TGF-β1 and AS progression

**Figure 3:**
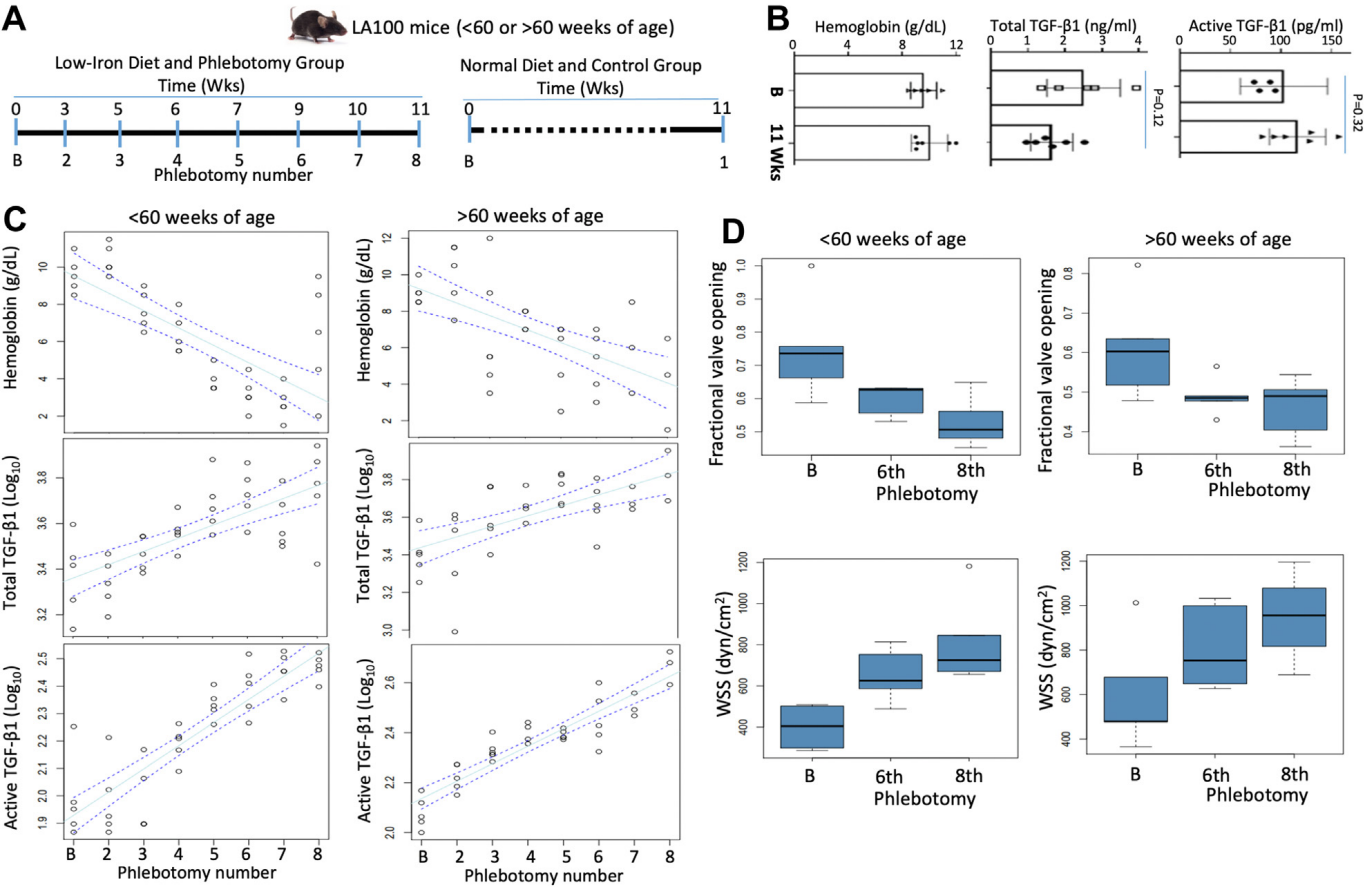
Anemia in LA100 Mice Is Associated With Higher Plasma Levels of Total and Active TGF-Β1 and AS Progression (A) Schematic diagram of anemia induction using low-iron diet and high-volume (300-500 μL) phlebotomy in <60 weeks) and >60 weeks of age LA100 mice. Control LA100 mice underwent low-volume (100 μL) phlebotomy. Weeks on diet and number of phlebotomies are indicated by blue bars. (B) Control LA100 mice <60 weeks of age were bled at low volume (100 μL) at baseline (B) and after 11 weeks. Hb levels in blood, collected at baseline (B) and at 11 weeks, were measured by HEMAVET HV950. Plasma total and active TGF-β1 levels were measured by enzyme-linked immunosorbent assay. (C) LA100 mice older and younger than 60 weeks were fed a low-iron diet followed by repeated high-volume (300-500 μL) phlebotomy up to 11 weeks as shown in A. Measurement of Hb levels at the indicated phlebotomy numbers showed progressive anemia with increasing phlebotomy numbers in both age groups (top). Plasma total and active TGF-β1 levels were measured by enzyme-linked immunosorbent assay, and both total and active TGF-β1 levels increase with the number of phlebotomies and correlated strongly with decreased Hb levels in both older and younger mice (*P <* 0.0001) (bottom 2 panels). (D) AS parameters of fractional valve openings and WSS in both younger and older mice were measured by echocardiography and found to be significant after the sixth and eighth phlebotomies compared with baseline (*P <* 0.001). Abbreviations as in Figures 1 and 2.

The observed inverse correlation between plasma total TGF-β1 and Hb levels in AS patients and in LA100 mice led us to consider whether anemia itself results in increased plasma TGF-β1 levels. To test this hypothesis, we induced anemia by administering an iron-deficient diet and performing 7 repeated large-volume (300-500 μL) phlebotomies at the times described in the Methods section and shown in Figure 3A for up to 11 weeks in 5 LA100 mice at 65 weeks of age and 5 LA100 mice at 25 weeks of age. A separate control group of LA100 mice (25 weeks) underwent low-volume (100 μL) phlebotomy only at baseline (pre) and after 11 weeks (Figure 3A). The low-volume phlebotomy did not produce any change in Hb or plasma TGF-β1 levels (both total and active) (Figure 3B). LA100 mice that underwent repeated large-volume (300-500 μL) phlebotomies developed progressive anemia with time, with Hb levels that were negatively correlated with the number of phlebotomies in both old and young mice (*P <* 0.0001) (Figure 3C). Plasma TGF-β1 levels (both total and active) increased progressively with phlebotomy number, as did anemia in both old and young groups (*P <* 0.0001) (Figure 3C). Anemia was associated with significant AS progression (ie, decreased fractional valve opening, increased aortic valve thickness and increased peak velocity, and wall shear stress) in both young and old LA100 mice after the sixth and eighth phlebotomies, respectively, compared with baseline (*P <* 0.001) (Figure 3D, Supplemental Figure 3).

### Platelets are major contributors of plasma TGF-β1 levels in the severe anemia model

**Figure 4:**
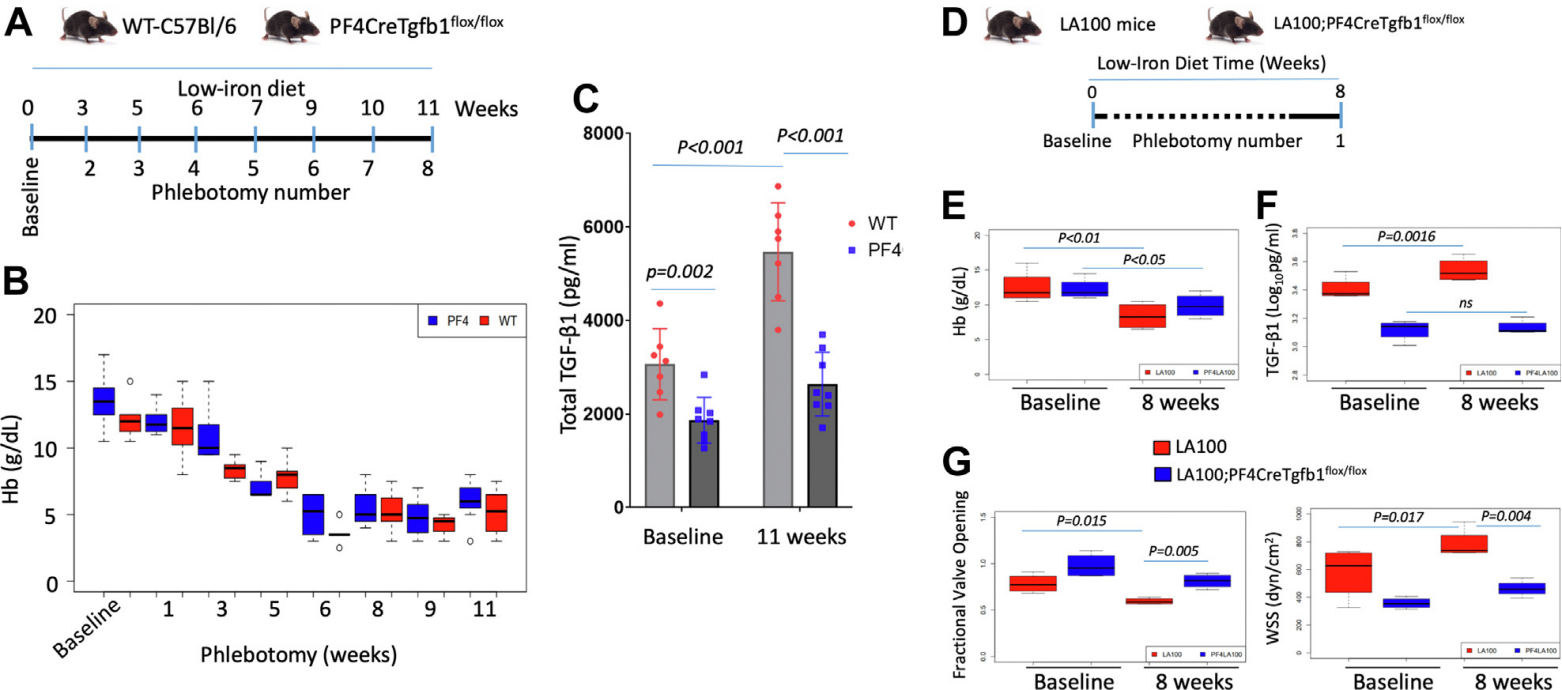
Anemia Increases Plasma Total TGF-Β1 in WT-C57Bl/6 Mice, But Not in Mice With Targeted Inactivation of Tgfb1 in Platelets (PF4CreTgfb1^flox/flox^) (A) Schematic diagram of the phlebotomy protocol for WT-C57Bl/6 and PF4CreTgfb1^flox/flox^ mice at 12 to 16 weeks of age fed a low-iron diet with large-volume (300-500 μL) phlebotomies are indicated by blue bars. (B) Assessment of Hb with subsequent phlebotomy demonstrated WT-C57Bl/6 and PF4CreTgfb1^flox/flox^ mice became anemic compared with their baseline levels (*P <* 0.001). (C) Plasma total TGF-β1 levels at baseline and at 11 weeks after successive phlebotomies were measured by enzyme-linked immunosorbent assay. (D) Schematic diagram of the low-iron diet-induced mild anemia protocol for LA100 and LA100;PF4CreTgfb1^flox/flox^ mice at 30 to 35 weeks of age fed a low-iron diet, and were bled low volume (100 μL) at baseline and after 8 weeks. (E) LA100 (30 weeks) and LA100;PF4CreTgfb1^flox/flox^ (35 weeks) mice were fed with low-iron diet for 8 weeks. Measurement of Hb levels after 8 weeks showed mild anemia. (F) Plasma total TGF-β1 levels were measured by enzyme-linked immunosorbent assay and TGF-β1 levels increased in LA100 mice *(P =* 0.0016), but not in LA100;PF4CreTgfb1^flox/flox^ mice (ns). (G) AS parameters of fractional valve opening and WSS to be significantly different between LA100 and LA100;PF4CreTgfb1^flox/flox^ mice after 8 weeks of anemia. Abbreviations as in Figures 1 and 2.

To evaluate the platelet contribution to the increased plasma TGF-β1 levels induced by phlebotomy, we induced anemia using low-iron diet and large-volume (300-500 μL) phlebotomies (Figure 4A) in a cohort of WT-C57Bl/6 mice (n = 7) and mice deficient in platelet TGF-β1 (PF4CreTgfb1^flox/flox^; n = 8).^22^ Hb levels decreased equally in both groups with phlebotomy (Figure 4B). In agreement with the data from our previous study,^22^ TGF-β1 levels were 35% to 50% lower in the PF4CreTgfb1^flox/flox^ mice at baseline and after anemia induction (Figure 4C). These data suggest that platelets are a major source of the increased TGF-β1 induced by phlebotomy.

### Loss of platelet TGF-β1 reduces AS progression in the presence of mild to moderate anemia

Whereas age-related anemia in both LA100 without phlebotomy and AS patients is in the mild to moderate range, we also tested the effect of loss of platelet TGF-β1 in the presence of anemia. We induced mild to moderate anemia by feeding a low-iron diet for 8 weeks without phlebotomy to both LA100 mice and LA100 mice with deficiency in platelet TGF-β1 (LA100;PF4CreTgfb1^flox/flox^) (Figure 4D). Average Hb levels decreased in both groups (from average 12.5 at baseline to 8.3 g/dL in 5 LA100 mice and 12.3 to 9.8 g/dL in 4 LA100;PF4CreTgfb1^flox/flox^ mice after 8 weeks of low-iron diet) (Figure 4E). In association with the development of anemia, TGF-β1 levels increased significantly in the LA100 mice after 8 weeks of low-iron diet (Figure 4F) (*P =* 0.0006). In contrast, TGF-β1 levels were 50% lower in the PF4CreTgfb1^flox/flox^ mice at baseline and after anemia induction (Figure 4F) (*P =* ns). The LA100 mice showed significant progression of both AS indicators during the 8 weeks of a low-iron-diet, whereas LA100;PF4CreTgfb1^flox/flox^ mice showed less AS progression despite these mice being 5 weeks older than control LA100 mice (LA100 mice had lower fractional valve opening [*P =* 0.005] and higher wall shear stress [*P =* 0.004] values at baseline than the LA100; PF4CreTgfb1^flox/flox^ mice) (Figure 4G).

### Producing anemia in LA100 mice induces severe acquired vWf syndromes

**Figure 5:**
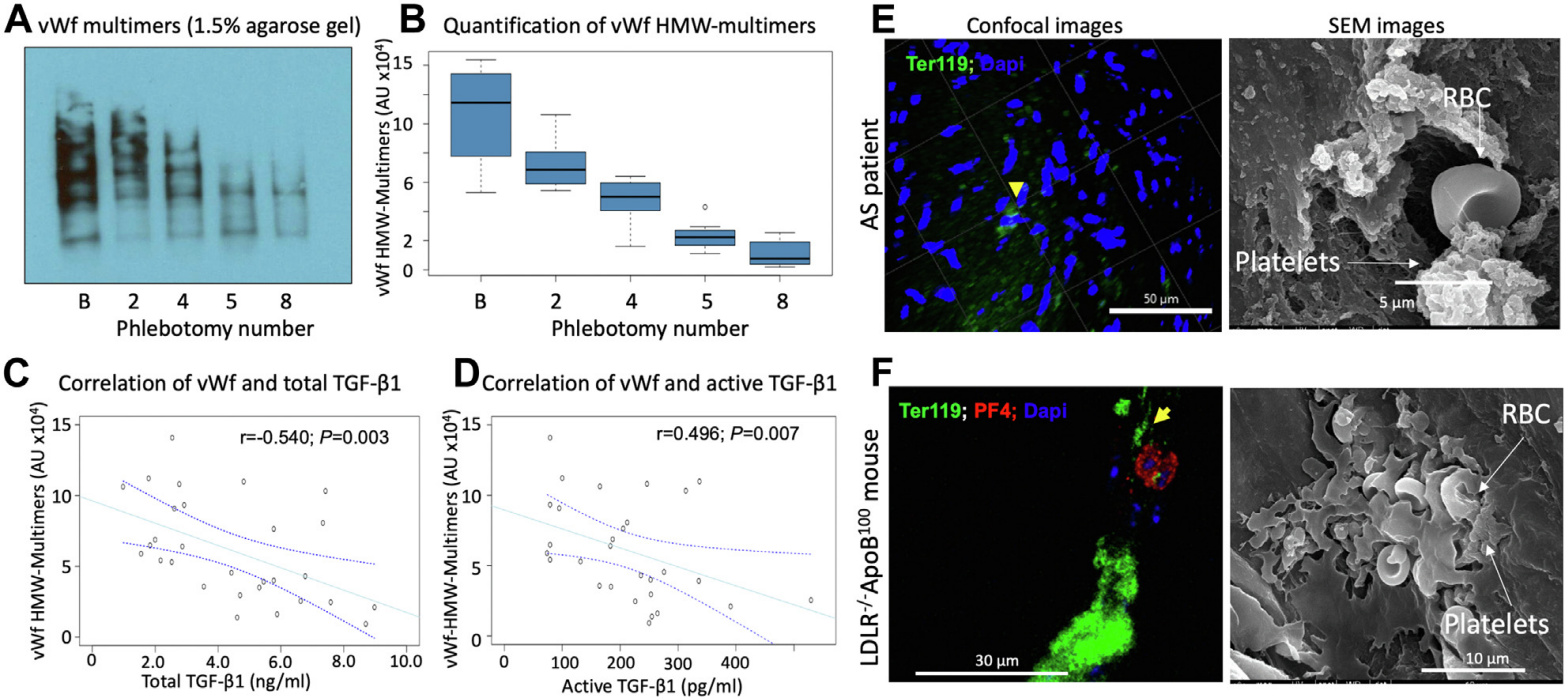
LA100 Mice Develop Anemia and AVWS (A) Representative nonreduced immunoblot of plasma von Willebrand factor (vWf) analyzed in 1.5% agarose gel showing gradual reduction of vWf multimers with increasing numbers of phlebotomies in an LA100 mice. (B) Quantification of high molecular weight (HMW)-vWf multimers in LA100 mice showing gradual decrease in HMW-vWf multimers as anemia progressed (*P <* 0.0001). (C, D) HMW-vWf multimers correlated negatively with total (r = −0.540; *P =* 0.003) and active TGF-β1 (r = −0.496; *P =* 0.007) levels in anemic LA100 mice. (E) Whole-mount human AS-patient valve stained for RBCs (Ter119-green) and valvular cells nuclei (DAPI-blue) Arrows in high-magnification whole-mount staining and scanning electron microscopy images of aortic valve show RBC inside the damaged valve surface associated with platelet aggregates. (F) Whole-mount of LA100 mouse aortic valve leaflets stained for RBCs (Ter119-green) and for platelets (PF4-red) and valvular cells nuclei (DAPI-blue). Arrows in high-magnification whole-mount staining and scanning electron microscopy images show RBC inside the damaged valve surface associated with platelet aggregates.

Since the acquired form of von Willebrand syndromes (AVWS) is characterized by a loss of the HMW vWf multimers, we quantified vWf multimer size in older LA100 mice before and after phlebotomy. We found a dramatic reduction of vWf multimer size as the mice became anemic, with almost complete loss of HMW vWf multimers after the eighth phlebotomy (Figures 5A and 5B and Supplemental Figure 4). We found negative correlations between HMW vWf multimers and both total and active TGF-β1 (Figures 5C and 5D). We also found a positive correlation between HMW vWf multimers and Hb levels (r = 0.561; *P =* 0.020).

### Activated platelets are physically associated with valvular endothelial cells

We imaged whole-mount aortic valves of AS patients and LA100 mice and found that platelet aggregates and RBCs attached to valvular cells (Figures 5E and 5F). Scanning electron microscopy imaging of the same whole-mount valves used for immunostaining confirmed these findings.

### LA100 mice expressing mutant C33S-TGF-β1 that cannot be activated by shear are partially protected from developing AS

**Figure 6:**
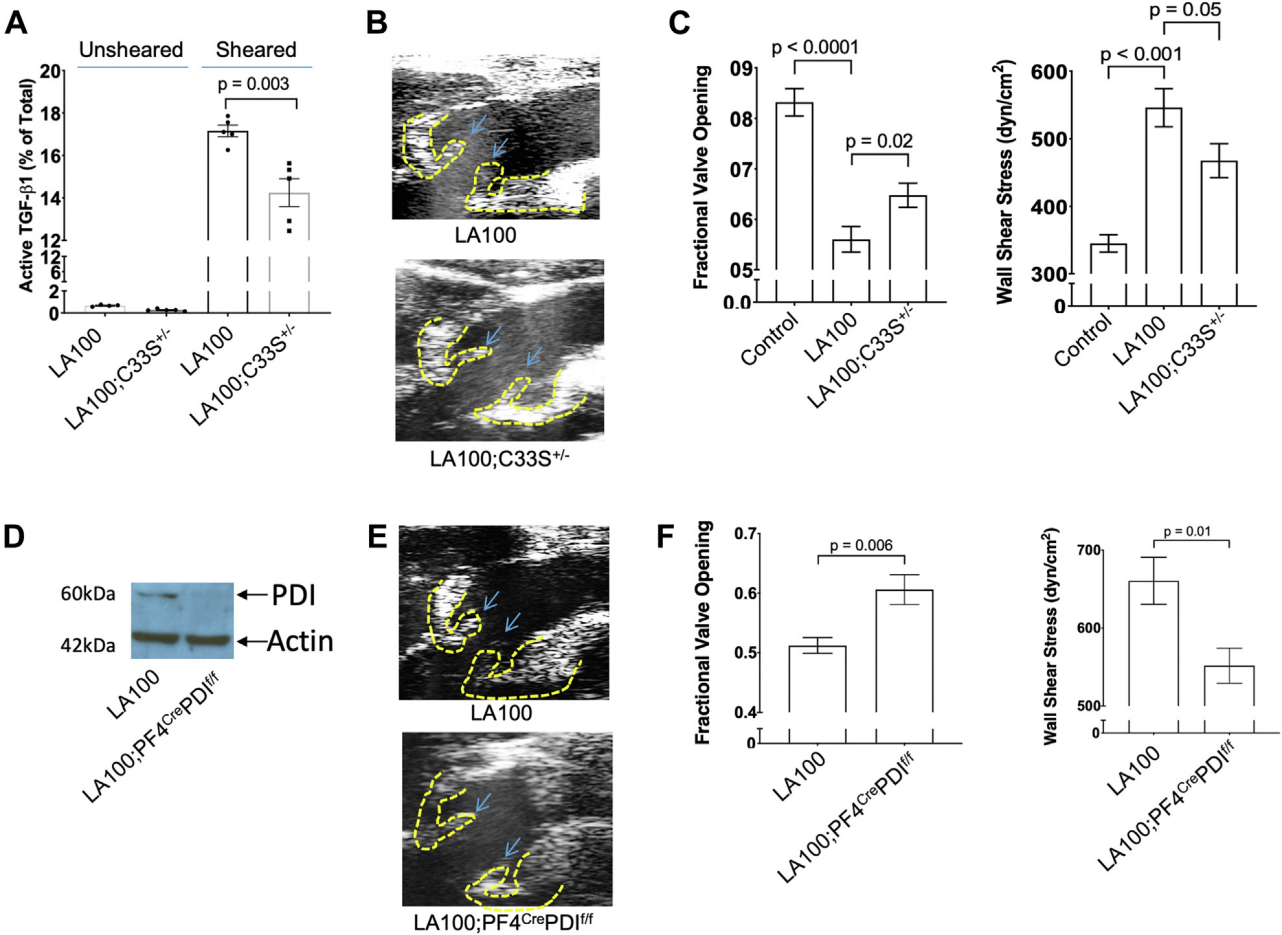
LA100;C33S^+/−^ and LA100;PF4CrePDI^flox/flox^ Mice Are Partially Protected From AS Progression (A) Active TGF-β1 levels in serum from control LA100 and LA100;C33S^+/−^ mice were measured by enzyme-linked immunosorbent assay after being subjected to shear rate of 1,800 s^−1^ for 2 hours. (B) Representative ultrasound images of aortic valve cusp (valve leaflets are marked with arrows) of LA100 and LA100;C33S^+/−^ mice. (C) Fractional valve opening and wall shear stress (WSS) were measured by echocardiography in control, LA100 and LA100;C33S^+/−^ mice >60 weeks of age. (D) Immunoblotting of platelet lysate from washed platelets with antibodies to protein disulfide isomerase (PDI) and actin showing PDI expression in platelets from WT and PF4CrePDI^flox/flox^ mice. (E) Representative ultrasound images of aortic valve cusps of control, LA100 and LA100;PF4CrePDI^flox/flox^ mice. (F) Fractional valve opening and WSS were measured by echocardiography in control LA100 and LA100;PF4CrePDI^flox/flox^ mice >60 weeks of age.

We previously demonstrated that latent TGF-β1 can be activated by shear, but that a mutant latent TGF-β1 protein that cannot form the disulfide bond with the LTBP-1 via its Cys33 residue because of a C33S mutation in the TGF-β1 precursor did not undergo shear-induced activation to the same extent as WT latent TGF-β1.^18^ To better assess the role of shear-induced TGF-β1 activation in AS progression, we generated LA100 mice expressing a heterozygous mutant TGF-β1 C33S allele (C33S^+/−^) (Supplemental Figure 1A). Serum-containing latent TGF-β1 from LA100;C33S^+/−^ mice older than 60 weeks of age produced ∼17% lower TGF-β1 activation by shear (17.16% in LA100 vs 14.25% in LA100;C33S^+/−^) than littermate control subjects (Figure 6A, Supplemental Figure 1B). They also showed significantly lower AS progression (Figure 6B) as measured by cusp separation (fractional valve opening) and wall shear stress (Figure 6C) compared with littermate LA100 mice. Aortic valves of these mice had lower collagen expression and p-Smad2 levels compared with littermate control LA100 mice (Supplemental Figures 1C and 1D).

### LA100 mice lacking PDI in platelets are partially protected from AS progression

We previously found that shear-induced activation of TGF-β1 involves thiol-disulfide exchange and PDI contributes to the thiol disulfide exchange and latent TGF-β1 activation in vitro.^21^ To assess the role of platelet-derived PDI in AS progression, we genetically depleted PDI expression in megakaryocytes/platelets by crossing LA100 mice with PF4CrePDI^flox/flox^ mice to generate LA100;PF4CrePDI^flox/flox^ mice as shown in Supplemental Figure 2.^23^ Immunoblotting of platelet lysates demonstrated that PDI levels were more than 90% lower (Figure 6D) in LA100;PF4CrePDI^flox/flox^ mice than littermate LA100 control mice. The latter mice also had less AS progression (Figure 6E) as measured by fractional valve opening and wall shear stress (Figure 6F).

## Discussion

Our data demonstrate that both LA100 mice, a mouse model that spontaneously develops AS, and patients with severe AS have higher plasma total TGF-β1 values compared with patient control subjects without valvular disease and healthy volunteers, and that TGF-β1 levels in AS patients correlate directly with age. Because AS is an age-related degenerative disease and its prevalence is ∼4% to 13% in patients >75 years of age, and because anemia is prevalent in AS patients older than 75 years^11^, we analyzed data in patient cohorts older than 75 years vs those 75 years of age or younger. Our findings are that: 1) AS patients older than 75 years of age have higher plasma TGF-β1 values and lower Hb levels than AS patients younger than 75 years of age or patient control subjects in either age group; and 2) there is a negative correlation between plasma TGF-β1 values and Hb levels in older, but not younger, AS patients. Because of these data, we induced anemia in LA100 mice to better define the potential impact of anemia on TGF-β1 levels and AS. We found that anemia in these mice was associated with higher TGF-β1 levels and with more rapid progression of AS. Moreover, mice with targeted deletion of platelet TGF-β1 were protected from AS progression and elevation of plasma TGF-β1 levels associated with induced anemia, either severe or moderate. We also studied the potential platelet contribution to AS progression by analyzing the impact of impairing the ability of latent platelet TGF-β1 to be activated by shear force because it is not bound to an LTBP-1.^18^ These mice also showed less AS progression compared with control subjects, as did mice with a targeted deletion of PDI from platelets. These data build on our previous studies showing the following: 1) shear force can activate latent TGF-β1 released from platelets in vitro;^18^ 2) plasma TGF-β1 levels increase over time in parallel with wall shear stress in the LA100 mouse model of AS;^23^ 3) megakaryocyte and platelet-derived TGF-β1 accounts for ∼45% of plasma TGF-β1 as judged by mice with targeted deletion of platelet TGF-β1;^22^ and 4) thiol-disulfide exchange mediated by PDI contributes to activation of TGF-β1.^18^^,^^21^

In trying to link the anemia to the development of increased TGF-β1 levels and AS progression, it is notable that anemia also led to altered wall shear stress,^31^ which may contribute to the release and activation of latent TGF-β1. One mechanism potentially linking anemia to increased wall shear stress is a compensatory increase in cardiac output (Supplemental Figure 5) and decreased systemic vascular resistance,^32^ because blood velocity is a key component of wall shear stress. Support for the physiologic significance of the increased shear stress was its correlation with loss of HMW vWf multimers, because shear stress alters the conformation of vWf, making it more susceptible to cleavage by ADAMTS-13.^33^ Together, these human and mouse data are in agreement with the report by Kearney et al. that anemia is an independent risk factor for progression to severe AS.^34^

**Figure 7:**
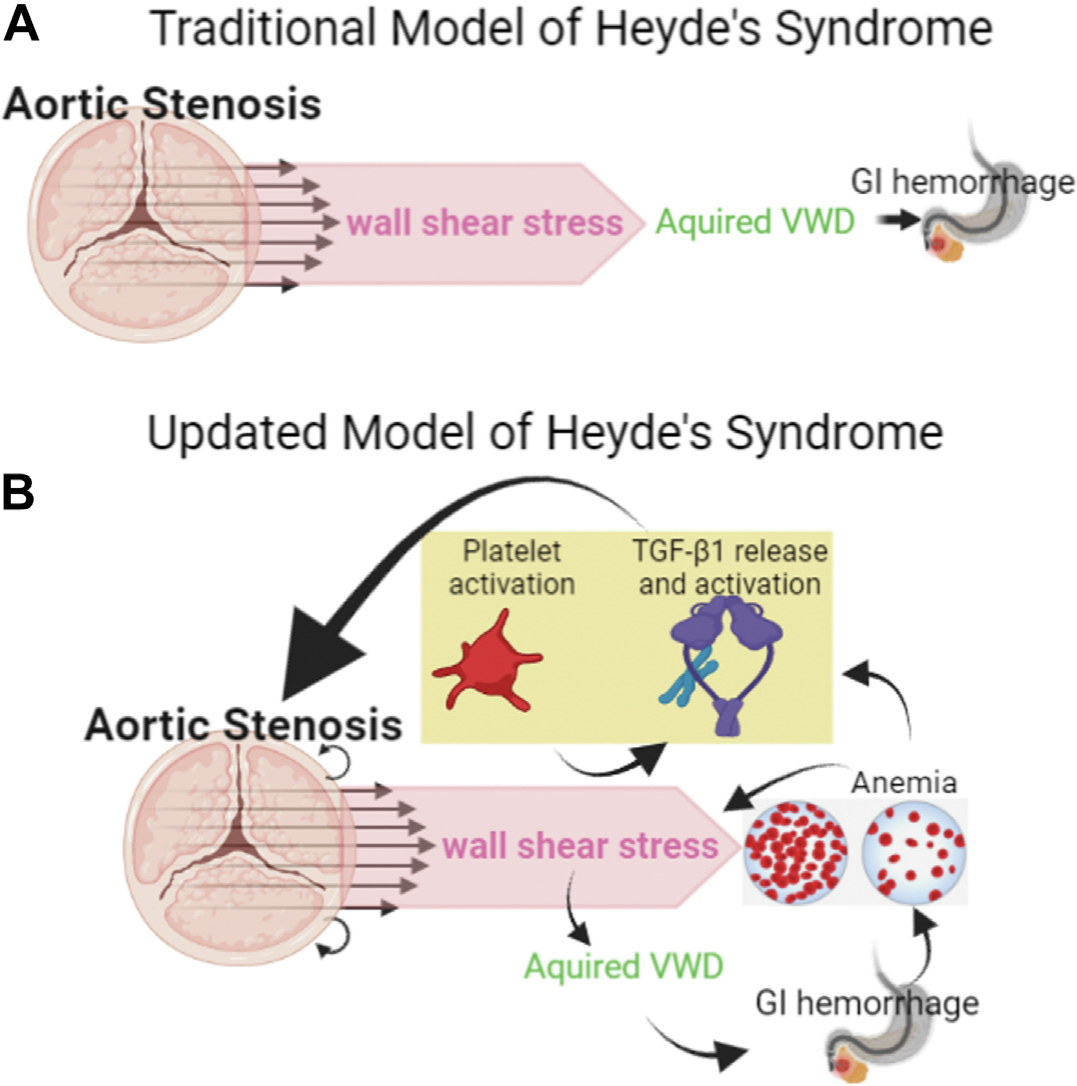
Heyde’s Syndrome Heyde’s syndrome has traditionally been depicted as the linear sequence depicted in A. Our new data indicate that anemia itself can feed back to increase active TGF-β1 levels in AS patients and LA100 mice, as depicted in B, which in turn, can increase AS severity in a vicious circle. Abbreviations as in Figure 1.

Our findings thus convert Heyde’s syndrome from a linear model consisting of AS leading to increased shear, leading to AVWS via unfolding of vWf and cleavage by ADAMTS-13,^33^ leading to gastrointestinal hemorrhage and anemia, to a vicious circle in which the anemia itself further increases shear, leading to both release of TGF-β1 from platelets and activation of released TGF-β1, resulting in further AS progression (Figure 7).

Both inherited and acquired forms of von Willebrand disease have been associated with vascular abnormalities, especially angiodysplasia of the gastrointestinal tract.^35^ It has been proposed that vWf interactions with endothelial cell αVβ3 inhibits signaling by VEGF through VEGFR2, and so loss of vWf results in abnormal proliferative angiogenesis. Inherited von Willebrand disease may, in addition, result in angiodysplasia via a mechanism related to loss of vWf in Weibel-Palade bodies.^35^ In fact, 2 of 10 anemia mice died and postmortem examinations revealed signs of bleeding in the gut as evident by whole mount immunostaining showing some areas with discontinuous blood vessels, RBC leakage, and fibrin deposition (data not shown). However, it is not clear whether the bleeding is a result of postmortem effects or spontaneous; thus, more work is needed to screen for angiodysplasia in older anemic mice with severe AS.

Our data provide a potential mechanism to account for the clinical observation of very rapid progression of AS as it reaches critical stenosis.^7^^,^^9^ This construct has potential therapeutic implications, because, if correct, anemic patients with AS may benefit from aggressive attempts to improve their anemia. Moreover, because the severity of the AVWS is a biomarker for increased shear stress, monitoring vWf multimers may provide a guide as to when the maximum benefit has been achieved by correcting the anemia. Our model also identifies plasma TGF-β1 as another potential biomarker of AS progression and inhibiting release of platelet TGF-β1 as a potential adjunct to slow the progression of AS. Future clinical studies will be required to test this hypothesis. Because valve replacement normalizes the shear gradient, it is expected to normalize TGF-β1 levels and the loss of HMW vWf multimers, which we plan to test in future studies. Since the current majority of valve replacement is by transcatheter aortic valve replacement, it would be interesting to compare TGF-β1 levels in patients before and after undergoing transcatheter aortic valve replacement vs surgical aortic valve replacement.

We recognize that in addition to gastrointestinal hemorrhage, intravascular hemolysis of RBCs as they traverse a stenotic valve at a very high shear stress may also contribute to anemia.^36^ This intravascular hemolysis may also result in the release of iron, which has been found to be deposited on aortic valves in association with calcification.^36^ If correcting anemia diminishes wall shear stress, it may have the added advantage of decreasing any ongoing hemolysis. Serum lactate dehydrogenase and plasma haptoglobin levels are valuable biomarkers of AS-associated hemolytic anemia.^37^

Anatomic support for a role for platelets comes from our finding of platelet aggregates in both human AS patient and LA100 mouse valve leaflets by whole mount immunofluorescent and scanning electron microscopy imaging. Our data are also consistent with previous observations that activated platelets are physically associated with valvular cells.^23^^,^^38^ In fact, Ozawa et al^39^ recently showed that LDLR^−/−^ (without ApoB100) mice lacking ADAMTS13 have increased aortic endothelial vWF, enhanced platelet adhesion, and evidence for TGF-β1 pathway activation in association with the development of hemodynamically significant AS. In patients with critical aortic stenosis, increased wall shear stress reduces large vWf multimers and leads to the development of acquired von Willebrand syndrome,^9^ which is also common in patients with left ventricular assist device implantation. It is postulated that both AS and left ventricular assist device generate high shear force, resulting in vWf unfolding and cleavage by ADAMTS13 or perhaps other mechanisms leading to impaired hemostasis.^40^ In fact, our previous study found an inverse correlation with platelet-released TGF-β1 and loss of vWf multimer size in heart failure patients implanted with LVAD.^26^

### Study limitations

First, the experimental anemia produced by both an iron-deficient diet and phlebotomy was more severe than the anemia experienced by patients with AS. To address this, we also tested animals subjected to more moderate anemia and found similar findings with regard to the protective effect of targeted loss of platelet TGF-β1. We did not study the potential contribution of other cells, including monocytes/macrophages, which can also secrete TGF-β1. Because both AS and anemia increase with age, other age-related metabolic and inflammatory conditions may also contribute to AS progression, and future studies are needed to address these issues. Our study is also limited by the relatively small AS patient sample size and the retrospective nature of the data collection. Similarly, the under-representation of women (n = 11, 20%) in this study limits its generalizability. We attempted to partially address these limitations by prospectively studying several mouse models in which we included more than 40% female mice in the analysis.

## Conclusions

We demonstrate that anemia, higher TGF-β1 levels, and AVWS are observed in older AS patients and LA100 mice, and the strong negative correlation between Hb and TGF-β1 and vWf multimers suggests that the anemia-induced increase in wall shear stress might be responsible for elevated activated TGF-β1 levels that then contribute to AS progression. This hypothesis has the potential to be tested clinically by studying the effect of aggressive correction of anemia in AS patients, perhaps using vWf multimers and/or TGF-β1 levels as biomarkers.

Perspectives **COMPETENCY IN MEDICAL KNOWLEDGE:** AS leads to increased shear force, which in turn leads to cleavage of high molecular weight vWf multimers, which in turn leads to a bleeding diathesis, gastrointestinal bleeding, and anemia (Heyde’s syndrome). Our data indicate that anemia itself may then accelerate AS progress via increasing shear-induced platelet-derived TGF-β1 release and activation, converting Heyde’s syndrome into a vicious circle that may account for the rapid progression of AS near the end of its course.

**TRANSLATIONAL OUTLOOK:** This proof-of-concept study demonstrates that anemia itself may contribute to AS progression via its impact on release and activation on TGF-β1. Thus, correcting anemia and targeting platelet-derived TGF-β1 release and activation may slow AS progression. Plasma TGF-β1 and vWf multimers may be valuable biomarkers of AS progression. Additional studies are required to test these hypotheses.

## Funding Support and Author Disclosures

This work was supported by grant HL148123 (to Dr Ahamed), in part by HL167656 (to Dr Ahamed), HL19278 (to Dr Coller), a Clinical and Translational Science Award (TR000043) from the National Center for Research Resources and the National Center for Advancing Translational Sciences (NCATS), and National Institutes of Health; and by funds from the Oklahoma Center for Adult Stem Cell Research, Presbyterian Health Foundation, Centers of Biomedical Research Excellence, and Stony Brook University. The authors have reported that they have no relationships relevant to the contents of this paper to disclose.
