# Supplementary material for: Evidence That Anemia Accelerates AS Progression Via Shear-Induced TGF-β1 Activation: Heyde's Syndrome Comes Full Circle
Source: JACC Basic Transl Sci. 2023 Nov 1;9(2):185–99. doi: 10.1016/j.jacbts.2023.09.007 (PMC10950403; doi:10.1016/j.jacbts.2023.09.007)
Supplement: Supplemental Figures 1-5 [file mmc1.pdf]

Supplemental Material

Supplementary Figures:

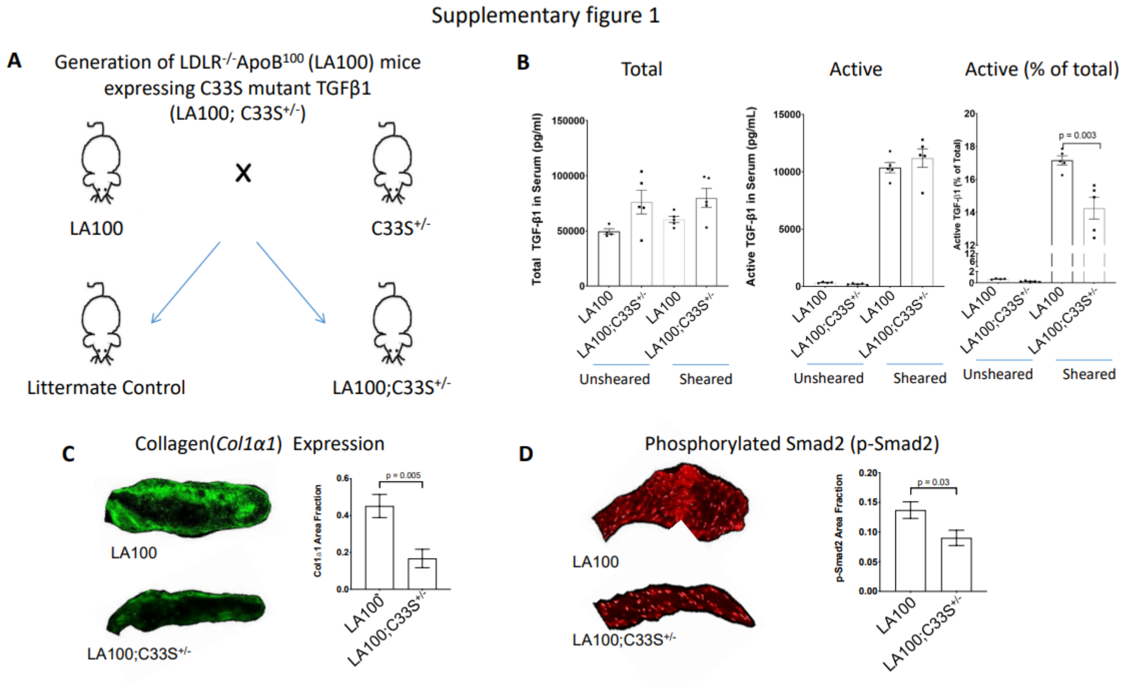

Supplemental Figure 1: (A) Schematic diagram of the breeding protocol to generate LDLR$^{-/-}$ApoB^{100} (LA100) mice expressing mutant C33S (LA100;C33S$^{+/-}$) mice and their littermate controls. **(B)** Total and active TGF-β1 levels in serum before and after shear for 2h were measured by ELISA active TGF-β1 levels were calculated as percent of total TGF-β1 levels. (**C**) Collagen and **(D)** p-Smad2 positive areas in the aortic valves of LA100 and LA100;C33S$^{+/-}$ mice as measured by Col1α1 p-Smad2 immunostaining and confocal imaging.

Supplementary figure 2

Generation of LA100 mice that lack platelet PDI (LA100;PF4^{Cre}PDI$^{flox/flox}$)

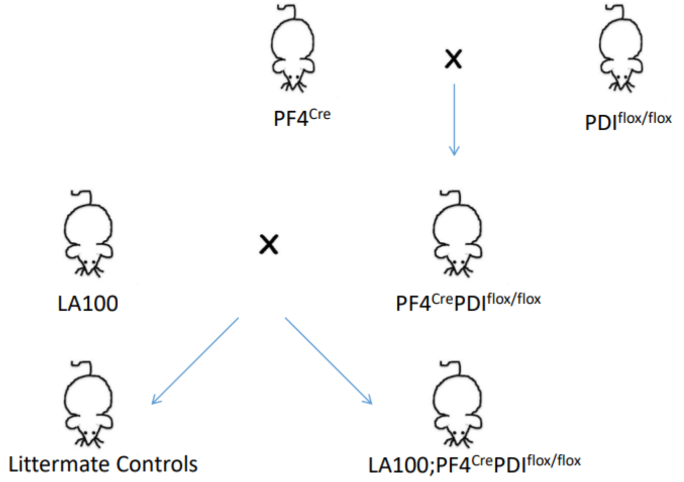

Supplemental Figure 2: (A) Schematic diagram of the breeding protocol to generate conditional deletion of PDI in platelets (LA100;PF4^{Cre}PDI$^{flox/flox}$) and their littermate controls.

Supplementary figure 3

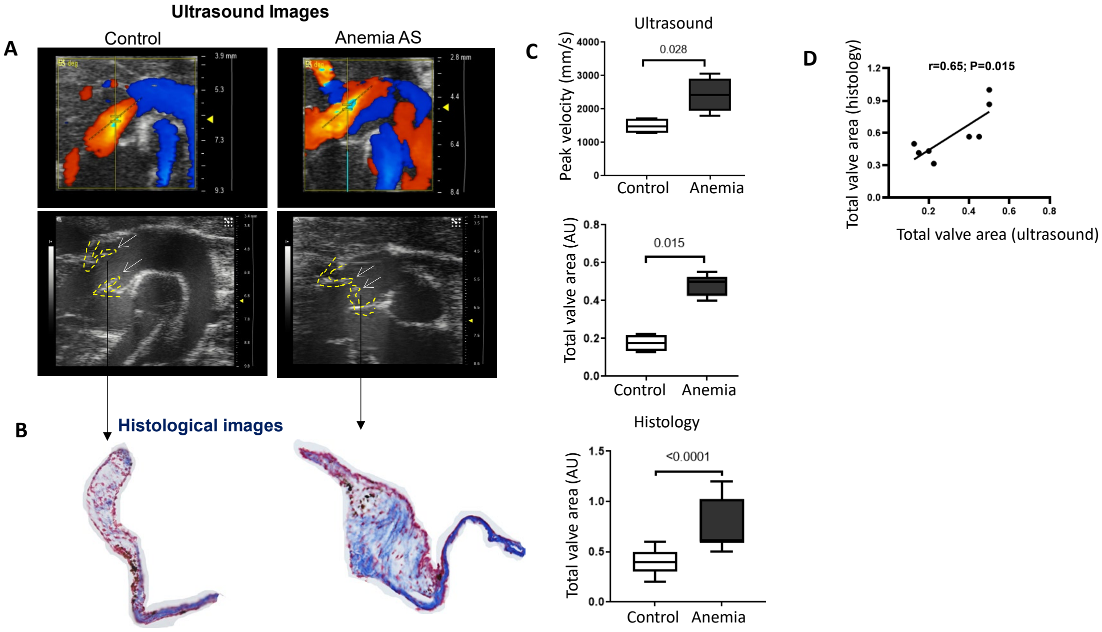

Supplemental Figure 3: (A) Representative ultrasound B-mode images with color doppler (upper panels) and aortic valve leaflets (lower panels, indicated by arrows) of control and anemia LA100 mice (n = 4-5), as measured by echocardiography. (B) Representative histological pictures of aortic valves leaflets of control and anemia LA100 mice. (C) Aortic peak velocity across the aortic valves of control and anemia LA100 mice, as measured by ultrasound with pulse-wave Doppler (upper panel); total valve area measured from echocardiographic B-mode images (middle panel); and from histological images (lower panel) (n = 4-5). (D) Pearson correlation of total valve areas measured from echocardiography and histology (r = 0.65; P= 0.015; n = 8).

Supplementary figure 4

Immunoblots of vWf multimers run on 1.5% agarose gels from different mice

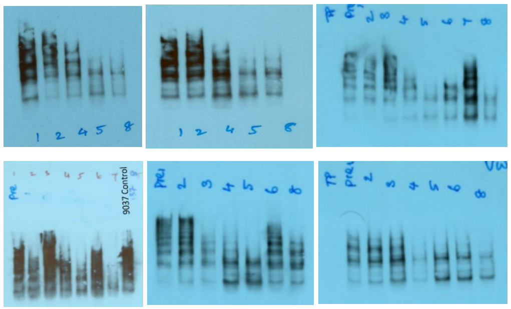

Supplemental Figure 4. Representative nonreduced immunoblots of plasma vWf analyzed on 1.5% agarose gels from 4 young and two old LA100 mice, showing gradual reduction of vWf multimers with increasing phlebotomies.

Supplementary figure 5

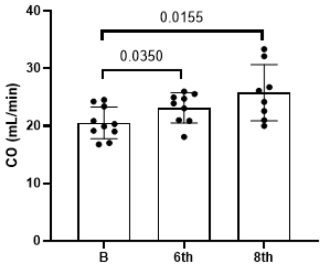

Supplemental Figure 5: Cardiac output (CO) in both younger and older LA100 mice were measured by echocardiography and found to be significant after the 6th and 8th phlebotomies compared to baseline (P<0.05).
